# Effects of Two-Week Betaine Supplementation on Apoptosis, Oxidative Stress, and Aerobic Capacity after Exhaustive Endurance Exercise

**DOI:** 10.3390/antiox9121189

**Published:** 2020-11-27

**Authors:** Ming-Ta Yang, Xiu-Xin Lee, Bo-Huei Huang, Li-Hui Chien, Chia-Chi Wang, Kuei-Hui Chan

**Affiliations:** 1 Center for General Education, Taipei Medical University, Taipei 110301, Taiwan; yangrugby@tmu.edu.com; 2 Department of Primary Care Medicine, Taipei Medical University-Shuang Ho Hospital, New Taipei City 23561, Taiwan; b101103133@tmu.edu.tw; 3 Charles Perkins Centre, School of Health Sciences, Faculty of Medicine and Health, The University of Sydney, Camperdown 2006, Australia; 1040609@ntsu.edu.tw; 4 Graduate Institute of Athletics and Coaching Science, National Taiwan Sport University, Taoyuan 333325, Taiwan; chienlihui@gmail.com; 5 Office of Physical Education, National Taipei University of Business, Taipei 10051, Taiwan; sunnywango@gmail.com

**Keywords:** lymphocytes, mitochondrial transmembrane potential decline, thiobarbituric acid reactive substance, protein carbonyl

## Abstract

This study evaluated the effects of 2 weeks of betaine supplementation on apoptosis, oxidative stress, and aerobic capacity after exhaustive endurance exercise (EEE). A double-blind, crossover, and counterbalanced design was adopted, with 10 healthy male participants asked to consume betaine (1.25 g of betaine mixed with 300 mL of sports beverage, twice per day for 2 weeks) or placebo (300 mL of sports beverage). All participants performed a graded exercise test on a treadmill to determine the maximal oxygen consumption (VO_2max_) before supplementation and then performed the EEE test at an intensity of 80% VO_2max_ after 2 weeks of supplementation. The time to exhaustion, peak oxygen consumption, maximal heart rate, and average heart rate were recorded during the EEE test. Venous blood samples were drawn before, immediately after, and 3 h after the EEE test to assess apoptosis and the mitochondrial transmembrane potential (MTP) decline of lymphocytes as well as the concentrations of thiobarbituric acid reactive substance and protein carbonyl. The results indicated that lymphocyte apoptosis was significantly higher immediately after and 3 h after EEE than before exercise in participants in the placebo trial. However, lymphocyte apoptosis exhibited no significant differences among the three time points in participants in the betaine trial. Moreover, apoptosis in the betaine trial was significantly lower immediately after and 3 h after exercise compared with the placebo trial. No differences were noted for other variables. Thus, 2 weeks of betaine supplementation can effectively attenuate lymphocyte apoptosis, which is elevated by EEE. However, betaine supplementation exhibited no effects on MTP decline, oxidative stress, or aerobic capacity.

## 1. Introduction

In a normal body state, the naturally occurring free radicals in humans have been reported to exert significant positive effects on immune system regulation [1] and to have a significant negative effect, namely peroxidation, on lipids, proteins, and DNA [2,3]. Exercise has been demonstrated to enhance muscular metabolism, and consequently, oxygen uptake, which further enhances the generation of free radicals and oxidative stress [4,5]. Apoptosis is a phenomenon that occurs when free radicals damage human DNA and cause accelerated programmed cell death [6]. One study indicated that the percentage of apoptotic cells increased significantly after running at an intensity of 80% maximal oxygen consumption (VO_2max_) until exhaustion, whereas it remained unchanged after running at an intensity of 60% VO_2max_ for an identical running time [7]. Therefore, in athletes, the higher the exercise intensity is, the more apoptosis occurs. Adequate apoptosis is an essential mechanism in the human body [8], but a high percentage of apoptosis can induce alterations in the physiology and viability of circulating leucocytes, which have a causal relationship with exercise-induced immune distress [9]. Nutritional interventions to attenuate inflammation and apoptosis may directly or indirectly benefit muscular recovery and subsequent performance [10].

Betaine, a natural compound, is commercially obtained from sugar beet [11]. It was first discovered in *Beta vulgaris* in the 19th century [12] and has been noted to be present in microorganisms, plants, and animals [13], with wheat, shellfish, spinach, and beetroot containing high levels of the compound [14,15]. Betaine can not only be absorbed by the human body from diet but also be converted from choline. Choline, the precursor of betaine, can be oxidized to betaine aldehyde by choline dehydrogenase [12]. The betaine aldehyde can be oxidized to betaine by betaine aldehyde dehydrogenase in the presence of NAD^+^ [16]. Therefore, the human body can also obtain betaine from foods rich in choline, such as eggs, meat, fish, and whole grains [17]. About 50% of choline in the intestine will be converted into betaine [18] and humans can obtain average 1 g of choline from daily diet [17]. As early as the 1990s, betaine was added to animal feed to evaluate its effects on growth performance [19,20] and disease prevention [21]. In the first study involving humans related to betaine supplementation and exercise performance, Armstrong et al. [22] observed that oxygen consumption during sprinting after acute betaine supplementation (5 g of betaine mixed in 1 L of carbohydrate–electrolyte fluid) was significantly higher than in those who consumed only carbohydrate–electrolyte fluid. However, betaine supplementation did not improve sprint performance in a hot environment. Furthermore, long-term betaine supplementation (1.25 g twice per day for 14 days) before an acute exercise session was noted to significantly increase the concentrations of growth hormone and insulin-like growth factor-1 as well as significantly decrease cortisol concentration [23]. Notably, a 14-day betaine supplementation was suggested to effectively promote protein synthesis. However, other studies have revealed that long-term betaine supplementation had no benefits on jump squat power, the number of bench press or squat repetitions [24], or the peak concentric or eccentric force outputs during isokinetic chest press [25]. Therefore, the effects of betaine supplementation on exercise performance remain unclear.

In addition to improving strength and power, betaine regulates organic osmolytes and protects the function of cells and mitochondria [12]. Therefore, some cell culture and animal studies have investigated the effects of betaine supplementation on apoptosis and oxidative stress in damaged cells [26,27,28,29]. A study by Veskovic et al. [26] observed that betaine decreased the liver’s expression of proapoptotic mediator Bax and increased antiapoptotic Bcl-2 in nonalcoholic fatty liver disease induced by a methionine–choline-deficient diet in mice. In addition, betaine increased superoxide-dismutase, catalase, glutathione peroxidase, and paraoxonase activities. Studies have suggested that betaine can effectively attenuate apoptosis and improve antioxidative defense. In addition, other studies have determined that betaine exerts antiapoptotic effects in human corneal epithelial cells [27] and antioxidative stress effects in the liver of rats [28,29]. Furthermore, our laboratory data revealed that a single dose of betaine supplement (1.25 g of betaine mixed in 300 mL of sports beverage) 1 h before an exhaustive endurance exercise (EEE) significantly decreased lymphocyte apoptosis but had no effects on mitochondrial transmembrane potential (MTP) decline [30].

Based on these aforementioned results, we hypothesized that long-term betaine supplementation attenuates apoptosis and oxidative stress induced by exercise and enhances aerobic capacity. Therefore, this study evaluated the effects of 2 weeks of betaine supplementation on apoptosis, oxidative stress, and aerobic capacity after EEE.

## 2. Materials and Methods 

### 2.1. Participants

Ten healthy male participants were recruited from National Taiwan Sport University, Taoyuan City, Taiwan. Individuals with diabetes and cardiovascular, renal, liver, or autoimmune diseases were excluded. All participants were requested to maintain their regular eating habits, avoid alcohol, and avoid consuming other nutritional supplements during the experimental period. The participants were informed of the requirements, benefits, and risks of the study before obtaining written informed consent. The study was approved by the Institutional Review Board of Fu Jen Catholic University, New Taipei City, Taiwan, with the IRB number C102053. The anthropometric data of participants were as follows: age: 24.60 ± 3.06 years; weight: 76.45 ± 9.58 kg; height: 177.00 ± 8.26 cm. Moreover, the performance in endurance exercise capacities of VO_2max_, 80% VO_2max_, and mean relative running velocity at 80% VO_2max_ were 50.21 ± 8.38 mL/kg/min, 40.20 ± 6.71 mL/kg/min, and 3.07 ± 0.26 m/s, respectively.

### 2.2. Experimental Design

**Figure 1:**
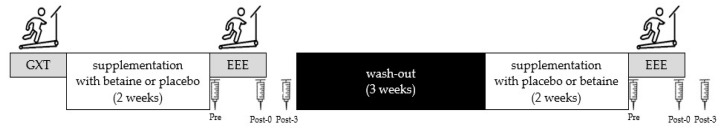
Experimental scheme. GXT, graded exercise test; EEE, exhaustive endurance exercise; Pre, before exercise; Post-0, immediately after exercise; Post-3, 3 h after exercise.

Regarding design, this was a double-blind, crossover, and counterbalanced study to evaluate the influence of 2 weeks of betaine supplementation on apoptosis, oxidative stress, and aerobic capacity after EEE in a healthy male population. The participants were asked to consume betaine or placebo, with at least 3 weeks of washout period between the trials. All participants performed a graded exercise test (GXT) until exhaustion on the treadmill to determine VO_2max_ before supplementation [31], and the speed equivalent to 80% VO_2max_ was implemented for an EEE test conducted on a treadmill for 30 min, after which speed was increased by 0.2 m/s every 1 min until exhaustion. The participants were instructed to consume a standard breakfast consisting of 648 kcal of total energy (66% carbohydrate, 11% protein, and 23% fat) 1 h before exercise. Blood samples were drawn before exercise (Pre), immediately after (Post-0), and 3 h after (Post-3) the EEE to determine the biomarkers of apoptosis and oxidative stress. Furthermore, plasma concentrations of betaine and choline before supplementation and at the Pre time point of EEE (after two weeks of supplementation) were also analyzed. In addition, the time to exhaustion, peak oxygen consumption, maximal heart rate (HR_max_), and average heart rate (AHR) were recorded during the exercise period. Figure 1 illustrates the scheme of the study.

### 2.3. Supplementation Protocol

The betaine supplementation strategy in the present study was based on the one suggested by Apicella et al. [23], wherein all participants consumed either 1.25 g of betaine (betaine powder; Twinlab, CO, USA) mixed with 300 mL sports beverage (Pocari Sweat, Otsuka, Taipei, Taiwan) or placebo (only sports beverage) twice per day for 2 weeks. The betaine for the supplementation was extracted from natural sugar beet and subsequently purified (99% pure). The sports beverage contains carbohydrates (66 g/L) and electrolytes of sodium (21 mEq/L), chloride (16 mEq/L), and potassium (4.9 mEq/L). The supplements for both trials had the same color and taste. The participants consumed supplements after breakfast and dinner during the experiment period.

### 2.4. Graded Exercise Test

The participants performed a GXT until exhaustion to determine the exercise intensity (80% VO_2max_). The treadmill (pulsar; h/p/cosmos, Nussdorf, Germany) GXT protocol was based on a previous study [31], wherein the treadmill speed started at 2.0 m/s and increased by 0.5 m/s every 4 min for the first three stages, after which the intensity was further elevated in increments of 0.5 m/s every 2 min until the participant reached exhaustion. Expired gas, VO_2_, and VCO_2_ were analyzed using gas analysis (Vmax Spectra 29c; SensorMedics, Yorba Linda, CA, USA), and HR was monitored (Polar S610; Kempele, Finland) at the same time points. Individual VO_2max_ was assumed to have been achieved when two of the following criteria were met: (1) respiratory exchange ratio greater than 1.1, (2) rating of perceived exertion greater than 18, and (3) HR within 15 beats/min of individual predicted HR_max_.

### 2.5. Blood Sampling and Analysis

Blood samples were drawn from the participants through antecubital vein suction into ethylenediaminetetraacetic acid tubes. A small portion of whole blood (400 μL) was then immediately analyzed for lymphocyte apoptosis and MTP decline in lymphocytes. Other blood samples were centrifuged at 2500× *g* for 20 min at 4 °C in an Eppendorf centrifuge 5804 R (Eppendorf AG, Hamburg, Germany). The supernatant was collected and stored at −20 °C before being analyzed for the concentrations of betaine, choline, thiobarbituric acid reactive substance (TBARS), and protein carbonyl (PC).

#### 2.5.1. Lymphocyte Apoptosis

The Annexin V-FITC apoptosis detection kit (BioVision, Milpitas, CA, USA) was applied to detect the lymphocyte apoptosis. After being gently mixed, 200 μL of the whole blood sample was transferred to a conical polypropylene test tube, and 4 mL of lysing buffer (Thermo Fisher Scientific, Waltham, MA, USA) was added. The solution was incubated at room temperature for 7 min before being centrifuged at 1500× *g* for 5 min at 20 °C, and the supernatant was then discarded. Subsequently, 3 mL of phosphate buffered saline (PBS) (Corning^®^, Corning, NY, USA) was added to wash the cells, the solution was centrifuged for 5 min, and the supernatant was then discarded. Lymphocytes were suspended with 300 μL of binding buffer. Thereafter, 3 μL of Annexin V-FETC and propidium iodide were added, and the solution was incubated in the dark at room temperature for 5 min. Finally, samples were gently drawn and then the subject to analysis by the BD FACSCalibur^TM^ flow cytometry (Becton Dickson, San Jose, CA, USA).

#### 2.5.2. MTP Decline in Lymphocyte

The MitoProbe™ JC-1 Assay Kit (Molecular Probes, Eugene, OR, USA) was applied to detect the MTP decline in lymphocyte. After being gently mixed, 200 μL of whole blood sample was transferred to a conical polypropylene test tube, and 4 mL of lysing buffer (Thermo Fisher Scientific, Waltham, MA, USA) was added. The solution was incubated at room temperature for 7 min before being centrifuged at 1500× *g* for 5 min at 20 °C, and the supernatant was then discarded. Subsequently, 3 mL of PBS (Corning^®^, Corning, NY, USA) was added to wash the cells, the solution was centrifuged for 5 min, and the supernatant was then discarded. Lymphocytes were suspended with 500 μL of PBS by using a MitoProbe^TM^ JC-1 assay kit, and then subjected to a dry bath in an incubator (MD-01N, Major Science, Taoyuan, Taiwan) at 37 °C for 15 min. Finally, samples were gently drawn and subjected to analysis by the BD FACSCalibur^TM^ flow cytometry (Becton Dickson, San Jose, CA, USA).

#### 2.5.3. Concentrations of Plasma Betaine and Choline 

**Figure 2:**
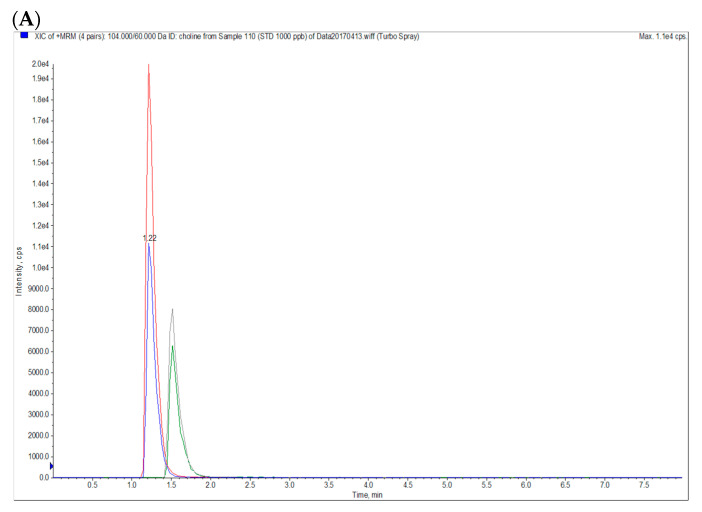

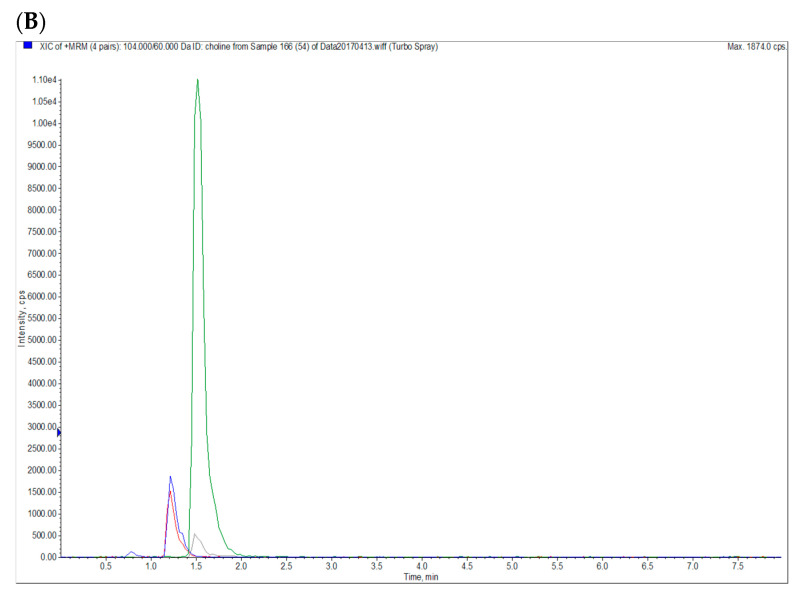
LC MS/MS Chromatograms of (**A**) betaine and choline standards (10 ng/mL each) and (**B**) a plasma sample. Blue: choline; Red: d_9_-choline; Green: betaine; Gray: d_11_-betaine.

To determine the blood concentrations of betaine and choline, d_11_-betaine and methyl-d_9_-choline were used as internal standards. Betaine hydrochloride, the standard of betaine, and d_11_-betaine were obtained from Chem Service (West Chester, PA, USA) and Cambridge Isotope Laboratories (Tewksbury, MA, USA), respectively. Choline chloride, the standard of choline, and methyl-d_9_-choline were purchased from Sigma-Aldrich (St. Louis, MO, USA). Initially, 30 μL of plasma was mixed with 90 μL of internal standard solution (10 ng/mL d_11_-betaine and d_9_-choline in methanol) in a microcentrifuge tube before being vortexed for 5 min and subsequently centrifuged at 5350× *g* for 10 min at 4 °C. The supernatants (60 μL) were placed in an LC-MS vial, and then analyzed using an AB SCIEX API 2000 liquid chromatography tandem-mass spectrometry system (Sciex Division of MDS, Toronto, ON, Canada). The analysis was performed using an Agilent 1260 Infinity Binary high-performance liquid chromatography system (Agilent Technologies, Santa Clara, CA, USA) equipped with an integrated degasser (G1322A), a pump (G1312C), an autosampler (G1329B), and a thermostat (G1330B). Analytes were chromatographically separated using an Waters XBridge BEH Amide column (100 mm × 2.1 mm, 3.5 μm) with a gradient mobile phase comprising (A) 0.1% formic acid (Sigma-Aldrich, St. Louis, MO, USA) in H_2_O, and (B) 0.1% formic acid in acetonitrile (Duksan, Seoul, Korea) under linear-gradient conditions (Period, A:B-%, *v*/*v*: 0–0.5 min, 30:70; 0.5–4 min, 30:70; 4–4.1 min, 50:50; 4.1–8 min, 30:70) at a flow rate of 400 μL/min; this procedure was a modification of that described by Bruce et al. [32]. The column was maintained at 28 °C. The monitoring conditions for multiple reactions are presented in Table 1, and Figure 2A,B present the LC MS/MS chromatograms of the standards and one blood sample.

#### 2.5.4. TBARS Concentration

TBARS concentration was analyzed using colorimetry, as described in a previous study [33]. Briefly, 200 μL of plasma was mixed with 200 μL of trichloroacetic acid (TCA; JT Baker, Phillipsburg, NJ, USA), 200 µL of Tris-HCl (Serva Electrophoresis, Heidelberg, Germany) and incubated at room temperature for 10 min. Thereafter, 400 μL of TBARS reagent, including 55 mM thiobarbituric acid (TBA) (from Merck, Darmstadt, Gemany) and 2 M sodium persulfate (Sigma-Aldrich Chemie GmbH, Steinheim, Germany) were added into the tube. Samples were kept to a dry bath in an incubator at 95 °C for 45 min and put on ice for 5 min. Subsequently, 400 μL of 75% TCA was added to the tube and mixed well, and the solution was then centrifuged for 5 min at 10,000× *g*. Thereafter, 100 μL of supernatant was loaded onto 96-well plates, and various concentrations of 1,1,3,3-tetraethoxypropane (Aldrich, St. Louis, MO, USA) were used to construct a standard curve, per the procedure described by Boadi et al. [34]. The absorbance was read at 530 nm using a Tecan Infinite M200 microplate reader (Tecan Austria GmbH, Grödig, Austria), and the result was applied to the regression formula to obtain the TBARS concentration.

#### 2.5.5. PC Concentration

PC concentration was analyzed using an enzyme-linked immunosorbent assay with a commercial assay kit (BioVision, Milpitas, CA, USA). Briefly, 100 μL of plasma was mixed with 10 μL of streptozocin solution, and the mixture was incubated at room temperature for 15 min before being centrifuged for 5 min; the supernatant was then transferred to a new tube. Subsequently, 100 μL of DNPH was added to each sample, which was then vortexed and incubated at room temperature for 10 min. Thereafter, 30 μL of TCA was added to each sample, which was then put on ice for 5 min, and centrifuged for 2 min. Then, the supernatant was removed, 500 μL of cold acetone was added to each tube, and the solution was placed in a sonicating bath for 30 s. Subsequently, samples were placed at –20 °C for 5 min before being centrifuged for 2 min, and the acetone was then carefully removed. These steps were repeated twice with 500 μL of cold acetone added. Finally, 200 μL of guanidine solution was added, and the solution was sonicated and incubated at 60 °C for 15 min. The 100 μL sample was loaded onto 96-well plates and read by the Tecan Infinite M200 microplate reader (Tecan Austria GmbH, Grödig, Austria) at an absorbance of 375 nm. The result was applied to the regression formula to obtain the PC concentration.

### 2.6. Statistical Analysis

Statistical analysis was performed using SPSS version 22.0 (IBM, Armonk, NY, USA). Data are expressed as mean ± standard deviation (SD). A paired *t* test was used to compare the variables of time to exhaustion, peak oxygen consumption, HR_max_, and AHR between the trials. A 2 (trials: betaine and placebo) × 3 (time points: Pre, Post-0, and Post-3) two-way repeated-measures analysis of variance was used to compare the variables of apoptosis, MTP decline, TBARS, and PC. When an interaction was noted, least significant difference post hoc tests were performed to determine where the difference occurred. The significance level was set at *p* < 0.05.

## 3. Results

### 3.1. Blood Concentrations of Betaine and Coline

**Figure 3:**
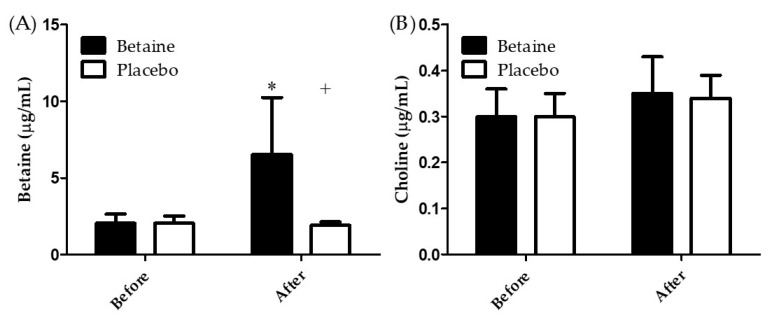
Blood (**A**) betaine and (**B**) choline concentrations before and after 2 weeks of supplementation. * Significant (*p* < 0.05) difference from before. + Significant (*p* < 0.05) difference from betaine trial.

Figure 3A illustrates the results of betaine concentrations in plasma before and after 2 weeks of betaine or placebo supplementation. A two-way repeated ANOVA with treatment and time indicated the interaction between two factors was significant (*p* < 0.05). The plasma betaine concentration in subjects in the placebo trial exhibited no significant differences before and after the supplementation. However, the plasma betaine concentration in the participants of betaine trial after 2 weeks of supplementation was significantly higher than before supplementation (6.53 ± 3.71 μg/mL vs. 2.04 ± 0.61 μg/mL, *p* < 0.05). Moreover, after 2 weeks of supplementation, the plasma betaine concentration was significantly higher for the betaine trial than the placebo trial (6.53 ± 3.71 μg/mL vs. 1.90 ± 0.23 μg/mL, *p* < 0.05). Figure 3B presents of choline concentrations in plasma before and after 2 weeks of betaine or placebo supplementation. A two-way repeated ANOVA with treatment and time indicated there is no interaction between two factors. The result shows that the supplementation strategy of present study can effectively enhance the betaine concentrations in plasma, rather than choline metabolism.

### 3.2. Effects of Betaine Supplementation on Lymphocyte Apoptosis and MTP Decline

**Figure 4:**
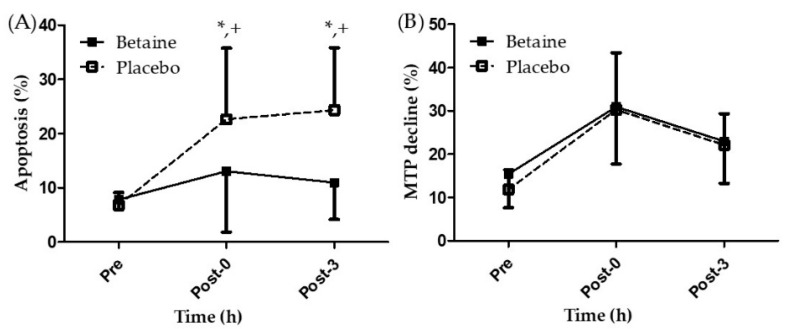
Changes of (**A**) lymphocyte apoptosis and (**B**) mitochondrial transmembrane potential decline in the exhaustive exercise after 2 weeks of betaine supplementation. * Significant (*p* < 0.05) difference from Pre. + Significant (*p* < 0.05) difference from betaine. Pre, before exercise; Post-0, immediately after exercise; Post-3, 3 h after exercise.

Figure 4A presents the results regarding lymphocyte apoptosis after 2 weeks of betaine or placebo supplementation. A two-way repeated ANOVA with treatment and time indicated the interaction between two factors was significant (*p* < 0.05). Lymphocyte apoptosis in participants undergoing 2 weeks of placebo supplementation was significantly higher at the Post-0 and Post-3 time points than at the Pre time point (22.61 ± 13.13%, 24.32 ± 11.49% vs. 6.77 ± 2.34%, respectively, *p* < 0.05). However, lymphocyte apoptosis exhibited no significant differences among the three time points in the betaine trial. Moreover, apoptosis in the betaine trial at Post-0 and Post-3 time points was significantly lower than that in the placebo trial. Figure 4B presents the results of the phenomenon of MTP decline after 2 weeks of betaine or placebo supplementation. No significant difference was observed in the phenomenon of MTP decline between the trials and time points (*p* > 0.05). The results indicated that twice per day supplementation of 1.25 g of betaine mixed in 300 mL of sports beverage for 2 weeks attenuated lymphocyte apoptosis immediately after EEE but can’t benefit on MTP decline.

### 3.3. Effect of Betaine Supplementation on Oxidative Stress

**Figure 5:**
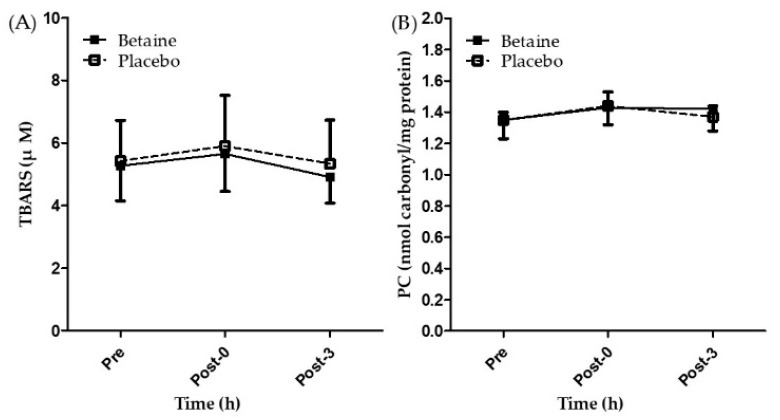
Changes of (**A**) TBARS and (**B**) PC in the exhaustive exercise after 2 weeks of betaine supplementation. Pre, before exercise; Post-0, immediately after exercise; Post-3, 3 h after exercise.

Figure 5A,B present the concentrations of TBARS and PC after 2 weeks of betaine or placebo supplementation. A two-way repeated ANOVA with treatment and time indicated there is no interaction between two factors in TBARS and PC. Furthermore, no significant differences were observed in the concentrations of TBARS and PC between the trials and time points (*p* > 0.05). The results showed that two weeks of betaine supplementation had no effects on biomarkers of exercise induced oxidative stress (TBARS and PC).

### 3.4. Effect of Betaine Supplementation on Aerobic Capacity 

All participants completed EEE, which involved running at an intensity of 80% VO_2max_ for 30 min on a treadmill, followed by a 0.2 m/s increase in speed every min until exhaustion. Table 2 indicates no significant differences between the trials regarding the time to exhaustion, peak oxygen consumption, HR_max_, or AHR (*p* > 0.05). The result shows that two weeks of betaine supplementation had no effects on the indicators of aerobic capacity (time to exhaustion, VO_2max_, HRmax, and AHR).

## 4. Discussion

This study examined the effects of 2 weeks of betaine supplementation on lymphocyte apoptosis, MTP decline, oxidative stress (TBARS and PC), and aerobic capacity (time to exhaustion, VO_2max_, HR_max_, and AHR) after EEE. The findings revealed that twice per day supplementation of 1.25 g of betaine mixed in 300 mL of sports beverage for 2 weeks attenuated lymphocyte apoptosis immediately after EEE. However, a 14-day betaine supplementation had no effects on MTP decline, oxidative stress, or aerobic capacity. In addition, the results revealed that the concentration of betaine in participants’ plasma increased significantly with 2 weeks of betaine supplementation. This result was consistent with a previous study that indicated 2 weeks of betaine supplementation (1.25 g of betaine mixed in 300 mL of sports beverage twice per day) can effectively enhance the concentration of betaine in plasma [24]. Moreover, no significant differences were noted in the concentration of choline in plasma between trials (Figure 3B). Therefore, the reason for the elevated betaine concentration in plasma with betaine supplementation was due to of the supplementation rather than choline metabolism. Therefore, the beneficial effects of attenuation of lymphocyte apoptosis after EEE can be attributed to the elevation in plasma betaine concentration. This study also revealed that 2 weeks of betaine supplementation could not reduce the production of TBARS and PC after EEE. The reactive oxygen species production is considered essential for exercise adaptations to occur [35], our result indicated the effectiveness of the betaine supplementation to reduce lymphocyte apoptosis is not via the redox processes.

The MTP is negative inside and positive outside under the normal status; however, when apoptosis is initiated through the depolarization of MTP, which opens the permeability transition pore of mitochondria, the increased membrane permeability of mitochondria results in MTP decline [36]. Therefore, mitochondria are believed to play a pivotal role in the intrinsic apoptosis pathway [37]. In addition, the extrinsic pathway is triggered by pro-inflammatory marker like tumor necrosis factor-α or by death receptors and ligands (Fas/ FasL complex) [38]. Therefore, apoptosis is induced by two distinct cell death pathways, either the intrinsic or extrinsic pathway [39,40]. This study showed that betaine supplementation could not reduce the production of TBARS and PC after EEE (Figure 4A,B), potentially because betaine supplementation is unable to effectively inhibit MTP decline after EEE. Craig [12] indicated that active coupled sodium and chloride ions and passive sodium ion independent transport systems promote the cellular absorption of betaine. Based on this finding, most studies regarding betaine supplementation have used betaine mixed in a sports beverage, with the sports beverage employed as the placebo [23,24,41,42,43]. Notably, sodium and chloride ions are essential electrolytes present in the blood and extracellular fluids. Moreover, sodium plays a crucial role in muscle contractions, and its depletion may affect the whole body [44]. Therefore, sodium and chloride ions, the essential ingredients of a sports drink, may have had an ergogenic effect on MTP, causing the consistent changes in MTP decline in both betaine and placebo trials. Hence, further studies should consider only betaine intake to evaluate its effects on MTP decline after EEE. In addition, this study revealed that 2 weeks of betaine supplementation can effectively attenuate lymphocyte apoptosis after EEE (Figure 3A). This result is consistent with in vitro studies, which have indicated that betaine can effectively decrease lymphocytes apoptosis in cell cultures [45,46]. Moreover, this result is consistent with a previous study in our laboratory that determined a single dose of betaine supplementation can effectively attenuate lymphocyte apoptosis immediately after EEE [30]. However, the present study revealed that 2 weeks of betaine supplementation can significantly attenuate lymphocyte apoptosis not only immediately after EEE but also up to 3 h after EEE. Therefore, the present study demonstrated that the ergogenic effect of 2 weeks of betaine supplementation is better than that of acute betaine supplementation. Lymphocyte apoptosis represents an extrinsic apoptosis pathway, which is initiated when the death ligands, such as tumor necrosis factor-α, bind to the death receptor [47]. Nevertheless, future studies might evaluate the inflammatory response after betaine supplementation to clarify the mechanism of lymphocyte apoptosis attenuation.

Animal studies have indicated that betaine can decrease oxidative stress [48,49]. Therefore, the present study hypothesized that betaine supplementation effectively attenuates oxidative stress caused by exhaustive exercise and enhances aerobic capacity. The present study did not observe an attenuation in lipid and protein peroxidation with 2 weeks of betaine supplementation. This result differs from that of previous studies involving cultured cells and animal tissue, which indicated that betaine can attenuate oxidative stress [48,49,50,51,52,53]. Halliwell [54] reviewed numerous studies with antioxidant paradox and concluded that the levels of oxidative damage measured in laboratory animals seem more responsive to being decreased by dietary antioxidants than they are in humans. This may one of the reasons with the inconsistent results. Moreover, the validity of measuring TBARS via colorimetry to be the indicator of lipid peroxidation is questionable [55]. This may the limitation of this study. Furthermore, some studies reported positive effects of betaine supplementation related to oxidative stress were conducted over an experimental period lasting more than 3 weeks [52,53,56]. More human studies with longer supplementation periods should be conducted to clarify the effects of betaine supplementation on oxidative stress.

The present study revealed that 1.25 g of betaine mixed in 300 mL of sports beverage twice per day for 2 weeks had no effects on the time to exhaustion, peak oxygen consumption, HR_max_, or AHR (Table 2). This finding is consistent with that of the previous study in our laboratory, which determined that acute betaine supplementation cannot enhance aerobic capacity [30]. Mitochondrial density can affect the consumption of oxygen during oxidative phosphorylation [57]. The fact that MTP decline is not suppressed by betaine supplementation may be one of the reasons for the lack of aerobic capacity enhancement. Furthermore, one study indicated that betaine supplementation effectively increases the force and power in participants who had a minimum of 3 months of prior resistance training [24]. And betaine supplementation had no effects on muscular strength in untrained participants [41]. The participants in the present study were healthy untrained men, which may explain why 2 weeks of betaine supplementation did not enhance aerobic capacity. Study of Schwab et al. [58] indicated that consuming 6 g per day of betaine for at least 4 weeks elevates plasma betaine concentration. However, other studies have revealed that consuming 6 g of betaine per day for 6 weeks and 12 weeks can increase risks to human health by elevating concentrations of low-density lipoprotein cholesterol and triglycerides as a consequence of increasing the concentration of homocysteine in blood [58,59]. Future studies should attempt to evaluate how various periods and dosages of betaine supplementation affect blood lipids to understand the effects of betaine supplementation on exercise.

## 5. Conclusions

Our study indicated that a 2 weeks supplementation of 1.25 g of betaine mixed with 300 mL of sports beverage twice per day significantly increased the plasma betaine concentration and effectively attenuated lymphocyte apoptosis after EEE. However, betaine supplementation had no effects on MTP decline, oxidative stress (TBARS and PC), or aerobic capacity (time to exhaustion, VO_2max_, HR_max_, and AHR). This study provides a nutritional supplementation strategy to attenuate exercise-induced lymphocyte apoptosis for intensive training athletes. Athletes may have better physiological status to conduct the training and shorten the recovery time during long term training periods. We recommend future studies use a higher dosage (more than 3 g, but less than 6 g per day) of betaine or combine it with other nutritional supplements to evaluate the effects of betaine supplementation on other athletic performance parameters or other related biomarkers of apoptosis.

## Figures and Tables

**Table 1:** Multiple reaction monitoring conditions of betaine and choline analysis.

Compounds	Parent Ion (m/z)	Molecular Ion (m/z)	DP (V)	FP (V)	EP (V)	CE (V)	CXP (V)
choline	104	60	28	320	8	26	7
d_9_-choline	113	69	35	367	8.8	26	8
betaine	118	58	35	350	10	38	6
d_11_-betaine	129	66	38	350	8	43	7

DP, declustering potential; FP, focusing potential; EP, entrance potential; CE, collision energy; CXP, collision cell exit potential.

**Table 2:** Changes in aerobic capacity after 2 weeks of betaine supplementation.

Trial	TTE (min)	HR_max_ (beats/min)	AHR (beats/min)	VO_2peak_ (mL/kg/min)
Betaine	33.61 ± 2.33	195.70 ± 7.06	173.33 ± 10.05	50.58 ± 6.50
Placebo	33.32 ± 1.62	195.70 ± 7.32	173.26 ± 8.99	47.40 ± 4.10

The data are presented as mean ± SD (*n* = 10). TTE, time to exhaustion; HR_max_, maximal heart rate; AHR, average heart rate; VO_2peak_, peak oxygen intake.
